# Medical collaboration enhances schoolteachers' support for children with neurodevelopmental disorders

**DOI:** 10.1002/pcn5.70171

**Published:** 2025-07-30

**Authors:** Kentaro Kawabe, Saori Inoue, Haruka Kozuki, Yu Matsumoto, Maya Kusunoki, Akiko Yamada, Saeko Sasaki, Masanori Isobe, Toshiya Murai, Shu‐Ichi Ueno, Fumie Horiuchi

**Affiliations:** ^1^ Department of Child Psychiatry Ehime University Graduate School of Medicine Ehime Japan; ^2^ Department of Neuropsychiatry, Molecules and Function Ehime University Graduate School of Medicine Ehime Japan; ^3^ Department of Psychiatry Kyoto University Graduate School of Medicine Kyoto Japan

**Keywords:** interpersonal conflict, medical collaboration, neurodevelopmental disorders, schoolteachers, stress

## Abstract

**Aim:**

Neurodevelopmental disorders (NDDs) present significant challenges for affected children and those responsible for their education. This study aims to examine the impact of medical collaboration with schoolteachers on the management of NDDs. It is essential to evaluate how structured collaborations may enhance educational support and reduce teacher stress.

**Methods:**

A web‐based anonymous survey was conducted from August to December 2023 among elementary and junior high school teachers across Ehime and Kyoto prefectures. Teachers completed an original questionnaire assessing their knowledge, attitudes toward students with NDDs, and responses to the Brief Job Stress Questionnaire (BJSQ). The survey categorized teachers based on their perceived effectiveness of medical collaboration. Logistic regression analysis, using 19 BJSQ subscales as independent variables, identified stress‐related factors associated with collaboration effectiveness.

**Results:**

A total of 812 valid responses were received, with 654 teachers reporting experience with medical collaboration. Among these, 249 teachers reported that collaboration was highly effective. The logistic regression analysis revealed that teachers who found medical collaboration effective had higher levels of qualitative job overload (odds ratio [OR]: 1.18, 95% confidence interval [CI]: 1.03–1.35, *p* = 0.02) and physical stress (OR: 1.04, 95% CI: 1.00–1.08, *p* = 0.04), but lower levels of interpersonal conflict (OR: 0.81, 95% CI: 0.71–0.92, *p* = 0.002).

**Conclusion:**

Teachers who perceived medical collaboration as effective reported lower levels of interpersonal conflict, suggesting a possible association between collaboration and reduced teacher burden. The findings support further integration of medical consultation in educational settings to better manage NDD‐related challenges.

## Graphical abstract

Medical Collaboration Enhances Teacher Support for Children with NDDs.

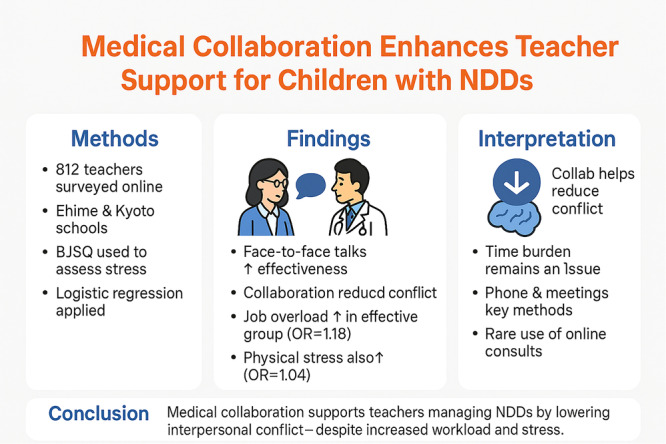

## INTRODUCTION

Neurodevelopmental disorders (NDDs) are characterized by developmental deficits and behavioral impairments in several areas of daily functioning, including personal, social, academic, and occupational domains.^1^ According to the Diagnostic and Statistical Manual of Mental Disorders—Fifth Edition (DSM‐5), NDDs are defined as a group of conditions that have their onset in the developmental period, leading to deficits that result in impairments of functioning.^2^ NDDs encompass intellectual disability, communication disorders, autism spectrum disorder (ASD), attention‐deficit hyperactivity disorder (ADHD), neurodevelopmental motor disorders (including tic disorders), and specific learning disorders (SLD). In Japan, the “Research About Children/Students with Probable Developmental Disabilities Who Need Educational Support in Regular Classes” has been conducted by the Ministry of Education, Culture, Sports, Science, and Technology (MEXT) every 10 years since 2002.^3^ This survey was carried out by teachers using questionnaires focused on ADHD, ASD, and SLD. According to the results of this survey, the estimated prevalence of these major disorders in elementary and junior high schools is as follows: ADHD at 4.0% (3.7%–4.3%), ASD at 1.7% (1.5%–1.9%), and SLD at 6.5% (6.1%–6.9%). The diagnosis of ASD has increased in Japan, as indicated by the National Database of Health Insurance Claims data.^4^ A formal diagnosis of ASD provides a range of benefits to families and children with ASD, particularly in high‐income countries, which is likely to increase the detection of ASD.^5^ Japan has a high‐quality healthcare system, including fully subsidized children's medical expenditures, and patients have the ability to visit any hospital. As a result, a majority of children with NDDs can visit hospitals if desired.

In Japan, education for children with various special educational needs is promoted based on the severity, overlap, and diversification of disabilities. Individualized educational support and instruction plans are created where necessary for children and students enrolled in regular classes. In addition to individual support in regular classrooms, special support education in elementary schools (generally for ages 7–12) and junior high schools (generally for ages 13–15) includes instruction in schools for special needs education, special classes, and special support classrooms/resource rooms. Adherence to an inclusive, rights‐based perspective is associated with a greater presence of students with NDDs, including ASD, in regular classrooms.^6^ Additionally, teachers' knowledge of NDDs is crucial for providing an adequate social and educational setting in which students with NDDs can be included.^7^ It is essential for teacher training to include the use of behavioral interventions for students with NDDs.^8^, ^9^

While the role of schoolteachers is important, it is well known that teaching is regarded as one of the most stressful professions, with long working hours.^10^ According to the results of the Organization for Economic Co‐operation and Development Teaching and Learning International Survey, an international, large‐scale survey of teachers conducted in 2018, the working hours of schoolteachers in Japan were the longest among the participating countries. The average weekly working hours of junior high school teachers in Japan were 56.0 h, significantly higher than the average of the participating countries, which was 38.3 h. Japan was also the only country in which the average working hours exceeded 50 h.^11^ Recently, the issue of emotional distress among schoolteachers has been gaining attention in Japan.^12^ Past studies have found a significant association between occupational stress (OS) and mental health,^13^, ^14^ as well as poor mental health and lower job satisfaction.^15^ OS is a psychological state that results from individuals' perceptions of an imbalance between job demands and their abilities.^16^ OS is consequentially known to produce negative organizational and extra‐organizational outcomes, such as low morale, poor performance, career uncertainty, absenteeism, health problems, work‐life conflict, turnover, and other reversals. It represents the primary risk factor for mental illness.^17^, ^18^

Interprofessional collaboration has become an essential component in the treatment of individuals with NDDs, as practitioners from a range of disciplines are often necessary to address the core features and co‐occurring conditions.^19^ Hence, collaboration between schoolteachers and doctors might be expected to result in the reduction of issues associated with NDDs. However, to the best of our knowledge, no study has examined the current state of schoolteachers' involvement in medical collaboration for NDDs. Therefore, this study aims to describe and analyze the impact of medical collaboration with schoolteachers for students with NDDs.

## MATERIALS AND METHODS

Participants were teachers from elementary and junior high schools throughout Ehime and Kyoto prefectures. A web‐based anonymous survey was conducted from August 2023 to December 2023. Teachers were invited to participate in the questionnaire survey via a letter. Ehime Prefecture is located in western Japan and had a population of 1,290,000 in 2023. There were 279 elementary schools and 129 junior high schools in Ehime, serving approximately 96,000 students. Kyoto Prefecture is located in central Japan and had a population of 2,500,000 in 2023. There were 353 elementary schools and 189 junior high schools in Kyoto, with about 180,000 students. A text message containing the link to the Google Form was shared with the teachers. The message included the study title, its aim, eligibility criteria for participation, potential advantages and disadvantages of participation, and an estimate of the average time required to complete the survey, which was approximately 20 min.

### Ethical considerations

This study was approved by the Institutional Review Board of Ehime University Graduate School of Medicine (IRB No. 2203001) and the Ethics Committee of Kyoto University Graduate School and the Faculty of Medicine (No. R3973‐1). Permission for this study was obtained from the boards of education in both Ehime and Kyoto prefectures prior to its implementation. Data were protected in accordance with the General Data Protection Regulation. The questionnaire was anonymized, and teachers responded via the web. Additionally, the first page of the Google Form clearly stated the informed consent requirements.

### Instruments

Teachers completed a questionnaire that included basic demographic information such as sex, age, teacher classification, experience in teaching special classes, and knowledge and attitudes toward students with NDDs. The questionnaire also assessed teachers' experiences with medical collaboration with healthcare facilities, as well as their responses to the Brief Job Stress Questionnaire (BJSQ).^20^ Teachers rated their experience with medical collaboration on a 4‐point scale, ranging from 1 (“I do not have experience with medical collaboration at all”) to 4 (“I often engage in medical collaboration every year”). In the subsequent analysis, teachers who selected “1” (no experience with medical collaboration) were excluded. Teachers were then asked about the methods used in medical collaboration and whether they felt burdened by it. Multiple response options were provided for each question.

### BJSQ

Psychological distress was assessed using the BJSQ to investigate job stressors (nine subscales, 17 items), stress responses (six subscales, 29 items), and modifiers (four subscales, 11 items). The items were rated on a 4‐point Likert scale, ranging from 1 (“strongly disagree”) to 4 (“strongly agree”). Higher scale scores indicate a higher level of psychological distress. The BJSQ has been widely used in research and practice in the field of mental health in the workplace in Japan.^21^ The English version of the BJSQ is available through the Ministry of Health, Labour, and Welfare, and its reliability and validity have been established at an acceptable level.^22^

### Statistical analysis

The study results are expressed as the mean ± standard deviation for continuous variables and as numbers and percentages for categorical variables. Teachers who had experienced medical collaboration were divided into two groups: those who felt that medical collaboration was effective and those who did not. The classification of medical collaboration as “effective” was based on teacher responses to a 4‐point Likert scale question. Responses of “4 = very effective” were classified as perceiving collaboration to be effective. Descriptive statistics were used to summarize the distribution of participants' characteristics. Chi‐square tests were used for categorical variables. Based on the basic information questionnaire, the effectiveness of medical collaboration was reclassified as a dependent variable. Effect sizes for between‐group differences were calculated using the φ‐coefficient from Chi‐square tests. Logistic regression analysis was conducted using the 19 subscales of the BJSQ as independent variables. All tests were two‐sided, and the significance level was set at 5%. All data were analyzed using SPSS Statistics software (version 23.0; IBM Corp., Armonk, NY, USA) for Windows.

## RESULTS

**Figure 1.**
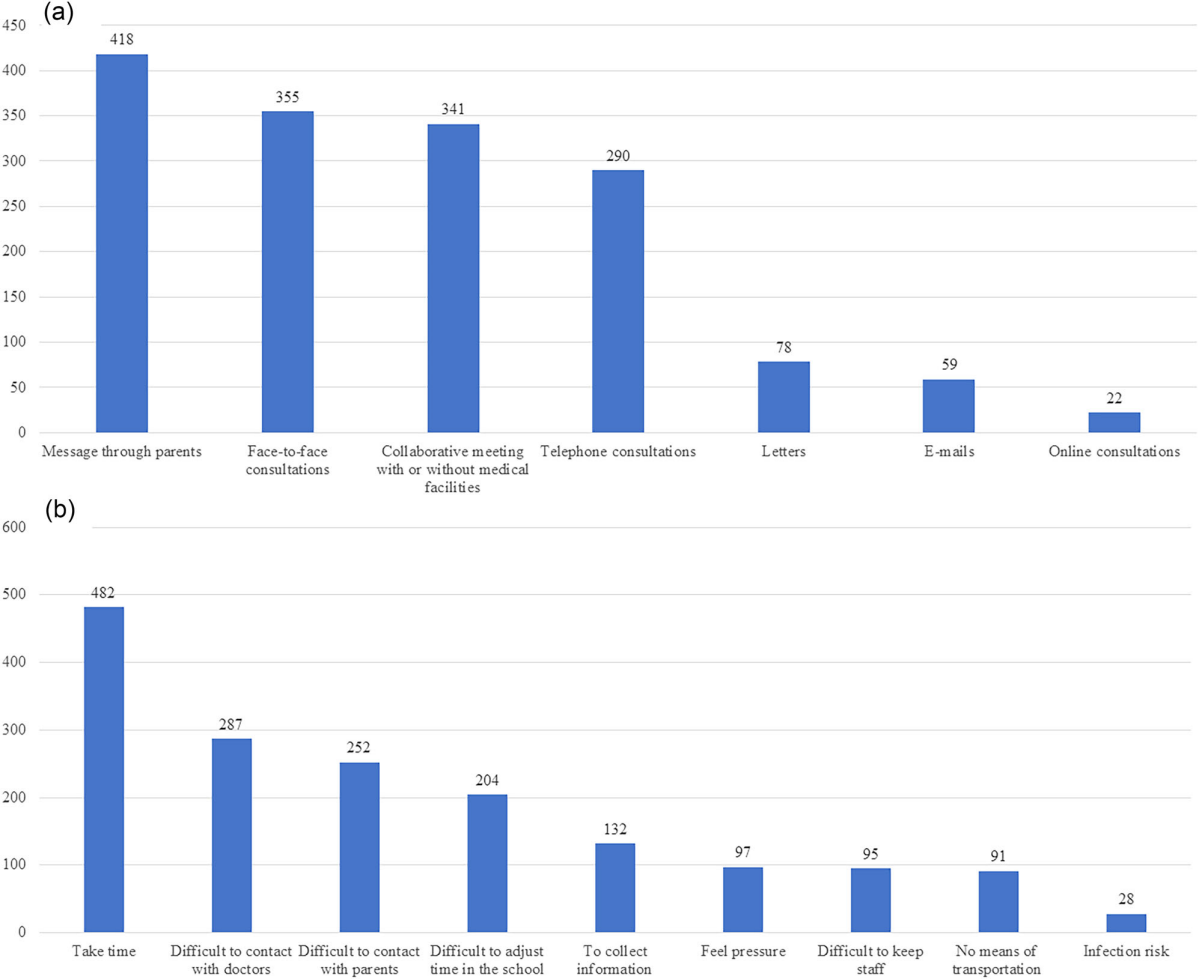
(a) Frequency of methods used in medical collaboration as reported by schoolteachers. (b) Perceived burdens of medical collaboration. Vertical axes represent the number of teachers who selected each item. The total number of respondents included in the analysis was *N* = 654.

Valid responses were obtained from 812 teachers. Teachers' demographics and characteristics are presented in Table 1. A little under 40% of teachers had interacted with students with NDDs. Of the teachers who had experience with medical collaboration, 654 participated, and 249 of them felt that the collaboration was very effective. The methods used for collaboration and a comparison of these methods between the two groups are shown in Figure 1 and Table 2. Teachers who felt the collaboration was effective used more face‐to‐face consultations and telephone consultations compared to the other group. Figure 1 shows the burden of medical collaboration for schoolteachers. The most significant burden for teachers was the time required for medical collaboration. Table 3 presents the results of the logistic regression analysis examining the factors associated with the effectiveness of medical collaboration and psychological distress in teachers. Teachers who found medical collaboration effective were associated with higher qualitative job overload (odds ratio [OR]: 1.18, 95% confidence interval [CI]: 1.03–1.35, *p* = 0.02) and physical stress (OR: 1.04, 95% CI: 1.00–1.08, *p* = 0.04), but lower interpersonal conflict (OR: 0.81, 95% CI: 0.71–0.92, *p* = 0.002). The Hosmer–Lemeshow statistic was X²(8) = 4.849, *p* = 0.774, suggesting a well‐calibrated predictive model. Multicollinearity among the 19 BJSQ subscales was assessed by calculating the correlation coefficients between all pairs of subscales in Table 4. All absolute correlation coefficients |r| were below 0.8, indicating that multicollinearity was not a significant concern in the model.

**Table 1:** Demographic characteristics of schoolteachers (*N* = 812).

Variables	Frequency (*n*)	Percentage (%)
Sex		
Male	320	39.4
Female	492	60.6
Age		
20–29 years	130	16.0
30–39 years	127	15.6
40–49 years	144	17.7
50–59 years	372	45.8
≥60 years	39	4.8
Classification of teachers		
Class teacher	323	39.8
Support teacher	60	7.3
Administrative teacher	154	18.9
Others	275	34.0
Experience of special class		
Yes	312	38.4
Years of experience		
≤3 years	61	7.5
4–9 years	138	17.0
10–19 years	138	17.0
20–29 years	190	23.4
≥30 years	285	35.1
Knowledge of NDDs		
Excellent	127	15.6
Very good	557	68.6
Fair	121	14.9
Poor	7	0.9
Experience of NDDs		
Excellent	614	75.6
Very good	170	20.9
Fair	27	3.3
Poor	1	0.1

Abbreviation: NDD, neurodevelopmental disorders.

**Table 2:** Group differences in the efficacy of the methods for medical collaboration (*N* = 654).

Methods of medical collaboration, *N*	Group that was effective, *n* = 249 *N* (%)	Group that was not effective, *n* = 405 *N* (%)	Effect size	*p* value
Messages through parents, 418	165 (66.3)	253 (62.5)	0.04	0.33
Face‐to‐face consultations, 355	165 (66.3)	190 (46.9)	0.19	<0.001
Collaborative meeting with or without medical facilities, 341	147 (59.0)	194 (47.9)	0.11	0.006
Telephone consultations, 290	139 (55.8)	151 (37.3)	0.18	<0.001
Letters, 78	37 (14.9)	41 (10.1)	0.07	0.07
E‐mails, 59	28 (11.2)	31 (7.7)	0.06	0.12
Online consultations, 22	13 (5.2)	9 (2.2)	0.08	0.04

*Note*: Effect sizes were used by φ‐coefficient in the chi‐square test.

**Table 3:** Logistic regression analysis examining the factors associated with the effectiveness of medical collaboration and psychological distress with teachers.

Subscale	B	OR	95% CI	*p*
Job stressors (9 subscales)				
Quantitative job overload	−0.12	0.88	0.78–1.01	0.07
Qualitative job overload	0.16	1.18	1.03–1.35	0.02*
Physical demands	−0.22	0.81	0.63–1.03	0.08
Job control	−0.02	0.98	0.88–1.10	0.76
Skill utilization	−0.01	0.99	0.76–1.28	0.91
Interpersonal conflict	−0.21	0.81	0.71–0.92	0.002**
Poor physical environment	0.03	1.03	0.84–1.27	0.78
Suitable jobs	−0.09	0.92	0.63–1.33	0.64
Meaningfulness of work	0.04	1.04	0.74–1.48	0.81
Stress responses (6 subscales)				
Vigor	−0.08	0.92	0.84–1.01	0.09
Anger‐irritability	−0.07	0.93	0.84–1.03	0.19
Fatigue	0.09	1.10	0.99–1.20	0.05
Anxiety	0.05	1.05	0.94–1.18	0.41
Depression	−0.07	0.94	0.88–1.00	0.07
Physical stress	0.04	1.04	1.00–1.08	0.04*
Modifiers (4 subscales)				
Supervisor support	0.01	1.01	0.90–1.14	0.86
Coworker support	−0.08	0.92	0.80–1.05	0.23
Support from family and friends	−0.04	0.96	0.86–1.08	0.50
Job and family life satisfaction	−0.14	0.87	0.70–1.07	0.18

Abbreviations: B, partial regression coefficient; CI, confidence interval; OR, odds ratio.

* *p* < 0.05

** *p* < 0.01.

**Table 4:** Correlation matrix of the 19 BJSQ subscales.

	Item 1	Item 2	Item 3	Item 4	Item 5	Item 6	Item 7	Item 8	Item 9	Item 10	Item 11	Item 12	Item 13	Item 14	Item 15	Item 16	Item 17	Item 18	Item 19
Item 1	–																		
Item 2	0.62	–																	
Item 3	0.43	0.44	–																
Item 4	0.21	0.10	0.09	–															
Item 5	−0.01	−0.05	−0.02	0.20	–														
Item 6	0.07	0.07	0.10	0.26	0.29	–													
Item 7	0.12	0.06	0.02	0.13	0.16	0.31	–												
Item 8	−0.02	−0.09	−0.04	0.31	0.29	0.20	0.15	–											
Item 9	−0.01	−0.08	−0.03	0.30	0.28	0.20	0.13	0.59	–										
Item 10	0.19	0.13	0.08	0.30	0.21	0.25	0.13	0.31	0.34	–									
Item 11	0.24	0.16	0.13	0.19	0.11	0.27	0.12	0.15	0.14	0.31	–								
Item 12	0.34	0.27	0.17	0.27	0.06	0.18	0.03	0.13	0.17	0.35	0.53	–							
Item 13	0.29	0.25	0.16	0.26	0.15	0.19	0.03	0.15	0.13	0.35	0.57	0.66	–						
Item 14	0.28	0.20	0.13	0.23	0.19	0.25	0.09	0.18	0.23	0.50	0.66	0.56	0.72	–					
Item 15	0.26	0.20	0.12	0.17	0.10	0.18	0.08	0.09	0.15	0.38	0.59	0.54	0.59	0.75	–				
Item 16	0.06	0.01	0.03	0.21	0.14	0.40	0.19	0.22	0.22	0.31	0.27	0.21	0.19	0.22	0.19	–			
Item 17	−0.02	−0.05	−0.03	0.19	0.15	0.39	0.21	0.24	0.27	0.28	0.18	0.13	0.16	0.19	0.17	0.68	–		
Item 18	0.04	0.03	−0.03	0.08	0.13	0.14	0.16	0.15	0.14	0.21	0.13	0.11	0.12	0.17	0.14	0.35	0.43	–	
Item 19	0.18	0.13	0.07	0.26	0.22	0.17	0.13	0.41	0.39	0.38	0.27	0.28	0.29	0.35	0.24	0.33	0.30	0.48	–

*Note*: Item 1: Quantitative job overload; Item 2: Qualitative job overload; Item 3: Physical demands; Item 4: Job control; Item 5: Skill utilization; Item 6: Interpersonal conflict; Item 7: Poor physical environment; Item 8: Suitable jobs; Item 9: Meaningfulness of work; Item 10: Vigor; Item 11: Anger‐irritability; Item 12: Fatigue; Item 13: Anxiety; Item 14: Depression; Item 15: Physical stress; Item 16: Supervisor support; Item 17: Coworker support; Item 18: Support from family and friends; Item 19: Job and family life satisfaction.

## DISCUSSION

### Key findings

This study examined teachers' knowledge of NDDs, their experiences with medical collaboration, and how such collaboration relates to various aspects of the teaching profession. Our results showed that only 38.4% of teachers felt they had useful collaboration between schools and medical facilities. Additionally, our findings indicated that effective medical collaboration can help reduce interpersonal conflict. However, despite the effectiveness of medical collaboration, teachers who experienced it reported higher qualitative workload and physical stress.

### Comparison with previous research

The experiences of school leaders with interpersonal conflict are an inevitable consequence of human interaction.^23^ The relationship between leadership and workplace stress can have negative outcomes for school effectiveness and school improvement, particularly in fostering positive staff relations.^24^ Teachers in leadership roles for students with autism may be more likely to experience interpersonal conflict and stress. According to self‐reports from Finnish elementary school principals, more than 35% of principals identified interpersonal conflicts as a significant contributor to their work‐related stress.^25^ In addition, a lack of support from superiors causes less inclusive and more negative attitudes toward students with NDDs and makes teachers less likely to provide appropriate learning environments.^26^ Realistically, the support of parents and students with NDDs, especially those who have emotional and behavioral problems, is necessary. Therefore, stress reduction for teachers is a high priority on a daily basis. Haydon et al. reported that protective factors for teacher stress included peer interaction, teacher perceptions, health and well‐being efforts, and administrative support. The effective management of stress involves supporting each other, getting along, being willing to collaborate, offering assistance, and maintaining a positive attitude.^27^

### Implications for practice

It has been shown that increased training, support, and time are key measures requested by the majority of teachers in order to achieve the successful inclusion of students with autism.^26^ However, the challenges teachers face in supporting children with NDDs are far‐reaching.^28^ A previous study reported that teachers who received consultation interventions had higher levels of self‐efficacy in teaching students with ASD.^29^ According to research on Italian teachers' attitudes toward ASD, the presence of a healthcare worker in the school environment is beneficial, and a multidisciplinary team is recommended for caring for students with NDDs.^30^ However, it is difficult for both teachers and medical doctors to find time for individual students.^31^ The diagnosis and care of NDDs require more time and resources, as they involve assessing both the child's behavioral and parental information.^32^ An international review of primary care doctors' consultation times showed that short consultation lengths were responsible for driving polypharmacy, overuse of antibiotics, and poor communication with patients.^33^ The field of child and adolescent psychiatry in Japan faces a shortage of child and adolescent psychiatrists, which necessitates multi‐professional support.^34^ Our study found that face‐to‐face consultations and telephone consultations were frequently identified as useful medical collaboration methods for schoolteachers. Telephone consultations offer flexibility and speed and eliminate the need for travel. Additionally, they provide many potential benefits, including mutual sharing of important information and consultation about students with NDDs, which can be considered a rudimentary form of telehealth.^35^ According to the results of this study, medical collaboration through online consultations was rarely conducted. Several studies have shown that hospitals and clinics observed a marked increase in telehealth services and a decrease in in‐person visits during the COVID‐19 pandemic.^36^, ^37^ Telehealth as a means of treating ASD has been expanding in the field of psychiatric clinics.^38^ As telehealth technology becomes increasingly widespread, there is a growing need for more research on its use in facilitating collaboration between schools and medical providers.

### Limitations and future directions

This study had some limitations. First, the study was based on an anonymous web questionnaire, and the sample size was limited. Additionally, many of the teachers in our sample exhibited a positive tendency toward education for students with NDDs, which limits the generalizability of the findings to all teachers. Second, there were no clinical criteria for students with NDDs in schools. Thus, further research is needed to explore strategies based on the type and severity of NDD traits. Third, the questionnaire was not strictly defined, and the findings rely on subjective responses from teachers. Therefore, discrepancies in responses may exist among teachers. In addition, the classification of medical collaboration as “effective” was based solely on subjective teacher ratings, without clearly defined criteria or thresholds. This may introduce interpretive bias and affect the consistency of reported outcomes. Fourth, this study did not rigorously define the content, duration, and number of staff involved in medical collaboration. The details of these factors may have varied across teachers.

There is a current and pressing need for medical collaboration to support schoolteachers. Interpersonal conflict has negative outcomes for teachers. Our findings suggest that teachers who perceived medical collaboration as effective also reported reduced interpersonal conflict.

## AUTHOR CONTRIBUTIONS

Kentaro Kawabe, Saori Inoue, and Haruka Kozuki designed the study concept, and Kentaro Kawabe wrote the first draft of the manuscript. All authors contributed substantially to the interpretation of the clinical data and approved the final version of the manuscript. Shu‐Ichi Ueno and Fumie Horiuchi supervised the study. All authors agree to be accountable for the content of this work.

## CONFLICT OF INTEREST STATEMENT

The authors declare that the research was conducted in the absence of any commercial or financial relationships that could be construed as a potential conflict of interest.

## ETHICS APPROVAL STATEMENT

This study was approved by the Institutional Review Board of Ehime University Graduate School of Medicine (IRB No. 2203001) and the Ethics Committee of Kyoto University Graduate School and the Faculty of Medicine (No. R3973‐1). The questionnaire was anonymized, and teachers responded via the web. Additionally, the first page of the Google Form included the informed consent requirement.

### PATIENT CONSENT STATEMENT

N/A.

### CLINICAL TRIAL REGISTRATION

N/A.

## Data Availability

The data that support the findings of this study are available from the corresponding author upon reasonable request.
